# Role of Nox2 and p22phox in Persistent Postoperative Hypertension in Aldosterone-Producing Adenoma Patients after Adrenalectomy

**DOI:** 10.1155/2016/2395634

**Published:** 2016-02-16

**Authors:** Xiaojing Geng, Li Yan, Jun Dong, Ying Liang, Yajuan Deng, Ting Li, Tongfeng Luo, Hailun Lin, Shaoling Zhang

**Affiliations:** ^1^Department of Endocrinology Medicine, Sun Yat-sen Memorial Hospital, Sun Yat-sen University, Guangzhou 510120, China; ^2^Department of Endocrinology Medicine, The Sixth Affiliated Hospital, Sun Yat-sen University, Guangzhou 510655, China; ^3^Department of General Internal Medicine, Sun Yat-sen University Cancer Center, Guangzhou 510060, China

## Abstract

Adrenal aldosterone-producing adenoma (APA), producing the salt-retaining hormone aldosterone, commonly causes secondary hypertension, which often persists after unilateral adrenalectomy. Although persistent hypertension was correlated with residual hormone aldosterone, the* in vivo* mechanism remains unclear. NADPH oxidase is the critical cause of aldosterone synthesis* in vitro*. Nox2 and p22phox comprise the NADPH oxidase catalytic core, serving to initiate a reactive oxygen species (ROS) cascade that may participate in the pathology. mRNAs of seven NADPH oxidase isoforms in APA were evaluated by RT-PCR and Q-PCR and their proteins by immunohistochemistry and Western blotting. NADPH oxidase activity was also detected. Nox2 and p22phox were especially abundant in APA. Particularly higher Nox2 and p22phox gene and protein levels were seen in APA than controls. Significant correlations between* Nox2* mRNA and* aldosterone synthase (CYP11B2)* mRNA (*R* = 0.66, *P* < 0.01) and Nox2 protein and baseline plasma aldosterone concentration (PAC) (*R* = 0.503, *P* < 0.01) were detected in APA; however, none were found between* p22phox* mRNA,* CYP11B2* mRNA,* p22phox* protein, and baseline PAC. Importantly, we found that Nox2 localized specifically in hyperplastic zona glomerulosa cells. In conclusion, our results highlight that Nox2 and p22phox may be directly involved in pathological aldosterone production and zona glomerulosa cell proliferation after APA resection.

## 1. Introduction

Primary aldosteronism (PA) is characterized clinically by autonomous aldosterone secretion from the adrenal gland, resulting in suppression of rennin secretion, hypokalemia, and hypertension. It is the most common cause of endocrinological hypertension [1]. Increased aldosterone levels are more prone to induce target organ damage [2] through genomic effects mediated by the mineralocorticoid receptor and possibly also by direct, nongenomic effects [3, 4]. Strong evidence exists to suggest that the cardiovascular and renal consequences of high blood pressure (BP) are more severe in patients with PA than age-, sex-, and blood pressure-matched essential hypertensives in retrospective case-control studies [5].

More than 50% of PA cases are known to be aldosterone-producing adenomas (APA, also known as Conn's adenoma) [6]. Several studies have shown that high serum aldosterone levels and hypokalemia in nearly all patients with APA are abolished after unilateral adrenalectomy. However, the prognosis of hypertension with APA is poor. Persistent hypertension occurs in 30% to 60% of cases after adrenalectomy [7, 8], and the molecular mechanisms responsible for this outcome are still ambiguous.

As a number of animal studies have associated oxidative stress induced by aldosterone with hypertension and heart failure [9, 10], it is likely that oxidative stress takes part in persistent hypertension after adrenalectomy. Maintenance of redox balance is essential for normal cellular functions. Increased reactive oxygen species (ROS) caused by any perturbation in this balance may lead to oxidative stress and cell dysfunction or death. NADPH oxidases (Noxs) have now been accepted as major sources for ROS in response to a wide range of stimuli, including angiotensin II, TNF, and IL-1*β*. They also render steroidogenic tissues acutely vulnerable to redox imbalance and dysfunction of macromolecules within the adrenocortical cells and other cells [11, 12]. Members of this oxidase family oxidize intracellular NADPH/NADH, allowing electron transport across the membrane and inducing molecular oxygen to superoxide anion (O_2_^•−^) and hydrogen peroxide (H_2_O_2_). The structure and function of Noxs have been primarily described in neutrophils and are composed of a membrane-bound complex (p22phox and Nox2) and cytosolic components (p47phox, p67phox, p40phox, and Rac1/Rac2) [13]. Nox2 and p22phox form the catalytic core of the Nox. Several homologues of Nox2 are now designated as part of the Nox family, including Nox1, Nox2, Nox3, Nox4, Nox5, Duox1, and Duox2 [13]. Notably, Nox2-derived ROS have recently been shown to increase aldosterone production in adrenocortical carcinoma cells [12].

To further improve our understanding of NADPH-induced oxidative stress involved in persistent hypertension after adrenalectomy in APA patients, we analyzed the clinical and pathological features and investigated the distribution and expression of Nox2 and p22phox in APA samples. Our findings provide a theoretical basis for prognostic evaluation and molecular target therapy in persistent hypertension.

## 2. Materials and Methods

### 2.1. Tissue Samples

Research protocols were approved by the Ethics Committee of Sun Yat-sen University (Guangzhou, China), and written informed consent was obtained from each patient. All patients were diagnosed with APA according to the guidelines of the Endocrine Society [14]. Nonfunctioning adenoma (NFA) was diagnosed in the absence of an abnormal adrenal steroid hormone secretion profile and obvious clinical syndrome of adrenocortical hyperactivity.

All adrenal tissues were freshly collected from patients undergoing adrenalectomy from July 2009 to September 2014 at the Sun Yat-sen Memorial Hospital, Sun Yat-sen University, and kept frozen in liquid nitrogen until further analysis. The study included 66 adrenocortical neoplasm tissue samples (APA 46; NFA 20) and 15 normal adrenal glands harvested from patients who, due to renal carcinoma or renal pelvic carcinoma, underwent adrenalectomy along with renalectomy. All tissues were each divided into two groups: 54 tissue samples (APA 29; NFA 14; normal adrenal tissues 11) for formalin embedding (subjected to histological examination and hematoxylin and eosin staining) and 28 tissues samples (APA 17; NFA 6; normal adrenal tissues 5) for RNA extraction, Nox activity assay, and Western blotting. Characteristics of patients from whom tissues were obtained for immunohistochemistry are shown in Table 1.

### 2.2. Nox Activity Assay

For protein extraction, the adrenal tissues were ground into powder in liquid nitrogen. The powdered tissue was then homogenized in 500 *μ*L lysis buffer, followed by centrifugation at 10,000 rpm for 30 min at 4°C. The lysates were collected, and the protein concentration was determined using the BCA Protein Assay Kit. The lysates were then used to evaluate Nox activity by lucigenin chemiluminescence using a commercial quantification kit (GENMED, Shanghai, China) according to the manufacturer's instructions.

### 2.3. RT-PCR and Quantitative Real-Time PCR (Q-PCR)

Total RNA from APA, NFA, and normal adrenocortical tissues was extracted using TRIzol reagent (Takara, Dalian, China) according to the manufacturer's instructions. cDNA was obtained by reverse-transcribing the total RNA with a cDNA synthesis kit (Takara). Expression levels of the following genes were examined:* Nox1*–*Nox5*,* Duox1*,* Duox2*,* p22phox*, and* CYP11B2*. CYP11B2 is the enzyme that catalyzes the final step of aldosterone synthesis, and its level represents the aldosterone level. The primer sequences used were designed by Takara (the primers, annealing temperature, and size of Noxs, CYP11B2, and GAPDH gene were shown in Supplemental Table  1 in Supplementary Material available online at http://dx.doi.org/10.1155/2016/2395634). PCR amplification from cDNA was performed in a reaction mixture with a final volume of 25 *μ*L. Cycling conditions were denaturation at 94°C for 30 s, annealing for 30 s at an appropriate temperature, and elongation at 72°C for 60 s. The optimal cycle number was 28 cycles. All PCR products were separated on a 2% agarose gel and visualized with ethidium bromide using UV light.

Q-PCR reactions were performed using the SYBR ExScript Q-PCR Kit (Takara). Amplification and analysis were performed on the Roche LightCycler 480 Real-Time PCR System. GAPDH was used as an internal control. Fold changes in target gene expression levels were calculated using the ΔCt method with the formula 2^(Ct(GAPDH)−Ct(target))^.

### 2.4. Western Blotting

Western blotting was carried out according to standard methods as described previously [15]. Briefly, adrenal gland tissue homogenates (30 *μ*g protein) were separated by 12% SDS-PAGE and transferred onto PVDF membranes. The membranes were incubated overnight at 4°C with primary antibodies, including rabbit anti-Nox2 (1 : 1000, ab31092, Abcam, Cambridge, MA, USA), rabbit anti-p22phox (1 : 1000, Ab75941, Abcam), and anti-GAPDH (1 : 1000, ab181602, Abcam). After being washed, membranes were incubated with anti-rabbit IgG-horseradish peroxidase- (HRP-) conjugated IgG (1 : 10000, Jackson ImmunoResearch Laboratories, West Grove, PA, USA) for 1 h at room temperature. The blots were exposed using a chemiluminescent detection system (Amersham Life Science, Buckinghamshire, UK) and quantified using National Institutes of Health Image software. GAPDH was used as the control.

### 2.5. Immunohistochemistry and Evaluation of Immunoreactivity

Immunohistochemical analysis using the Dako Envision detection kit (Dako, Glostrup, Denmark) was performed in accordance with the manufacturer's instructions as described previously [16]. Specimens containing both tumors and normal tissues were previously fixed in 10% buffered formalin and embedded in paraffin. Formalin-fixed, paraffin-embedded tissue sections of 4 *μ*m thickness were deparaffinized with xylene, rehydrated in serial dilutions of ethanol, and boiled for antigen retrieval. Blocking of endogenous peroxidase was performed by incubating tissue sections in 3.0% hydrogen peroxide for 15 min at 37°C. Sections were then incubated with the primary antibody for Nox2 (1 : 100, ab31092, Abcam) and p22phox (1 : 200, ab75941, Abcam) at 4°C for overnight. Sections were then incubated with an HRP-conjugated secondary antibody for 30 min at 37°C and then stained with 3,3′-diaminobenzidine (DAB). Hematoxylin was used for counterstaining. Finally, the sections were photographed with an Olympus BX51 microscope (Olympus, Tokyo, Japan).

A semiquantitative estimation was made using a composite score obtained by adding the values of the staining intensity (3+, strong staining; 2+, moderate staining; 1+, faint staining; and 0, negative) and the relative abundance of the positive cells (determined as 0–5%, 6–25%, 26–50%, 51–75%, and 76–100%). Using these criteria, the immunostaining results were evaluated blindly by three independent senior pathologists. For cases with a discrepancy, a consensus was reached during a common evaluation session.

### 2.6. Histological Examination

Adrenal changes of normal adrenocortical tissues, APA, NFA, and peritumoral adjacent tissues were observed by histological examinations performed on 4 *μ*m sections stained with hematoxylin-eosin-saffron (HES). Nuclei are stained in blue with hematoxylin, and the cytoplasm is stained in pink with eosin. The peritumoral cortex was defined as the entire cortex from glands harboring an APA. The main criteria used to determine zona glomerulosa (ZG) hyperplasia were based on published criteria [3].

### 2.7. Statistical Analyses

All continuous variables were expressed as the mean ± standard deviation (SD). Descriptive analysis included a median value and range. Statistical significance was evaluated by Fisher's exact test or the Mann-Whitney *U* test between different groups. Differences between more than two groups were assessed with the Kruskal-Wallis test. Correlations were determined using Spearman's correlation coefficient. Statistical analysis of the data was conducted using SPSS version 17.0, and *P* values < 0.05 were considered statistically significant.

## 3. Results

### 3.1. Clinical and Endocrinologic Features of Patients

In this study we compared clinical and endocrinologic features of 29 patients with APA and 14 patients with NFA (Table 1). APA patients had a higher baseline plasma aldosterone concentration (PAC, *P* < 0.01), baseline aldosterone-to-renin ratio (*P* < 0.01), and levels of urinary aldosterone (*P* < 0.01), urinary K^+^, and urinary Na^+^ than NFA patients. Interestingly, patients with APA required much more antihypertensive medication than patients with NFA. By contrast, lower baseline plasma renin activity (*P* < 0.01) and serum K^+^ (*P* < 0.01) were observed in APA patients, who were also younger (44 versus 53 years, *P* < 0.05), had smaller tumors (*P* < 0.05 and *P* < 0.01, resp.), and had higher systolic (*P* < 0.01) and diastolic (*P* < 0.01) blood pressure readings.

### 3.2. Nox2 and p22phox Expression and Distribution

**Figure 1:**
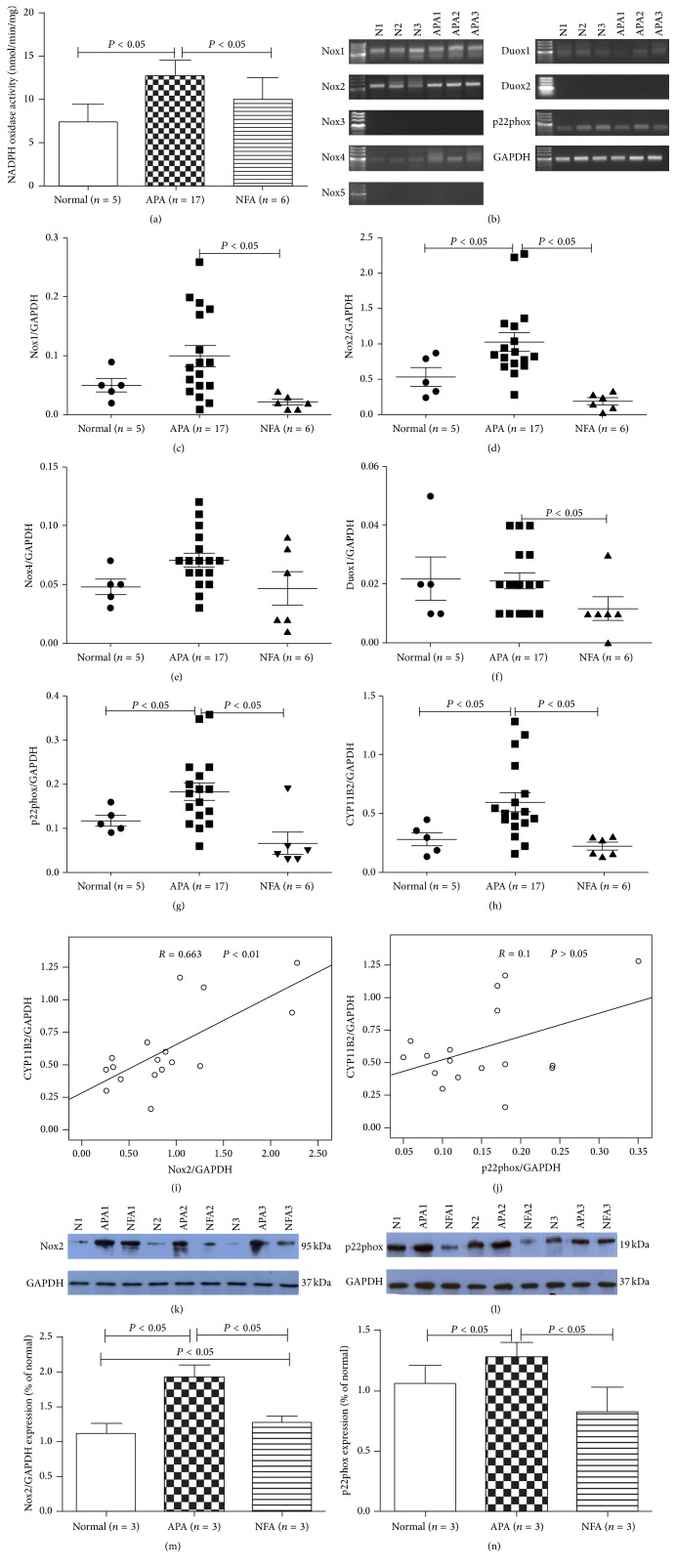
Activity and expression of Nox in different adrenal tissues. (a) Nox activity was detected in normal adrenocortical tissue, APA, and NFA by lucigenin chemiluminescence. (b) Expression of* Nox1*–*Nox5*,* Duox1*,* Duox2*, and* p22phox* mRNA in normal adrenocortical tissue and APA by RT-PCR. Signals for* Nox1*,* Nox2*,* Nox4*,* Duox1*, and* p22phox* were detected in normal adrenal tissue and APA. (c–h) Relative expression of* Nox1*,* Nox2*,* Nox4*,* Duox1*,* p22phox*, and* CYP11B2* in normal adrenocortical tissue, APA, and NFA measured by Q-PCR.* Nox2*,* p22phox*, and* CYP11B2* were primarily expressed in APA. (i-j) Correlation between* CYP11B2* mRNA with* Nox2*,* CYP11B2*, and* p22phox* mRNA in APA. A positive correlation between* Nox2* and* CYP11B2* mRNA was found. (k–n) Western blot analysis of Nox2 and p22phox in normal adrenocortical tissue, APA, and NFA. Nox2 and p22phox were enhanced in APA compared with normal adrenocortical tissue and NFA. Results of densitometric analysis of Nox2 and p22phox proteins normalized to GAPDH were shown.

To gain insight into the sequence of events linking Nox to aldosterone biosynthesis, we evaluated Nox activity using lucigenin chemiluminescence in normal adrenal tissues, APA, and NFA. Nox activity was higher in APA (Figure 1(a)). Moreover, mRNAs of* Nox1*,* Nox2*,* Nox4*,* Duox1*, and* p22phox* were found to be expressed in normal adrenal tissues, while those of* Nox3*,* Nox4*, and* Duox2* were negligible by RT-PCR analysis (Figure 1(b)). The mRNA expression levels of* Nox1*,* Nox2*,* Nox4*,* Duox1*,* p22phox*, and* aldosterone synthase* (*CYP11B2*) relative to* GAPDH* were also evaluated by Q-PCR. Compared with normal adrenal tissue and NFA, respective expression levels in APA of* Nox2* (1.9-fold and 2.6-fold),* p22phox* (1.6-fold and 2.8-fold), and* CYP11B2* (2.1-fold and 2.6-fold) were predominantly higher (Figures 1(c)–1(h)). Notably, the* Nox2* mRNA level showed a positive correlation with* CYP11B2* mRNA in APA patients (Figure 1(i)). However, the association was not statistically significantly different between* p22phox* and* CYP11B2* mRNA (Figure 1(j)). Furthermore, protein levels of Nox2 and p22phox were also higher in APA than normal adrenal tissue (1.8-fold and 1.6-fold, resp.) and NFA (1.2-fold and 1.4-fold, resp.) (Figures 1(k)–1(n)). Protein levels of Nox2 and p22phox were similar.

**Figure 2:**
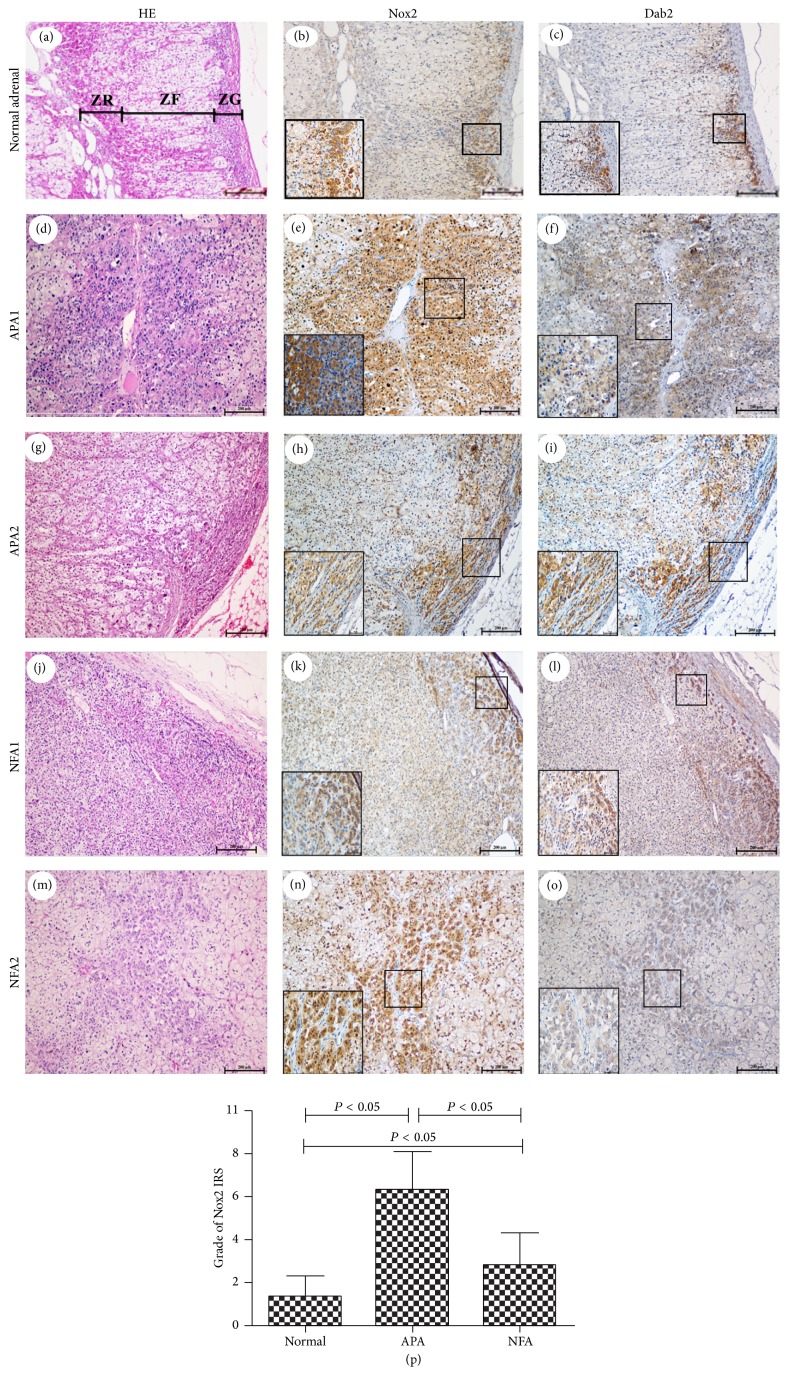
Pathological and immunohistochemical findings of Nox2 in normal adrenocortical tissue, APA, and NFA. (a, d, g, j, and m) HES staining of normal adrenocortical tissue (a), APA (d and g), and NFA (j and m). (b and c) Nox2 and Dab2 were expressed in normal adrenocortical ZG cells. However, Nox2 was also restricted to the ZR. (d–o) Nox2 and Dab2 expression were detectable and visible beneath the capsule (h, i, k, and l) and along the vasculature (e, f, n, and o) in APA and NFA as indicated by HES. Moreover, higher magnification revealed predominant cytoplasmic localization of Nox2 and Dab2. (p) Graphical illustration of statistical distributions of Nox2 in normal adrenocortical tissue, APA, and NFA. The Nox2 IRS (immunoreactive score) was higher in APA compared to those in normal adrenal tissue and NFA. Bar, 200 *μ*m on low magnification (100x) and 50 *μ*m on high magnification (400x).

**Figure 3:**
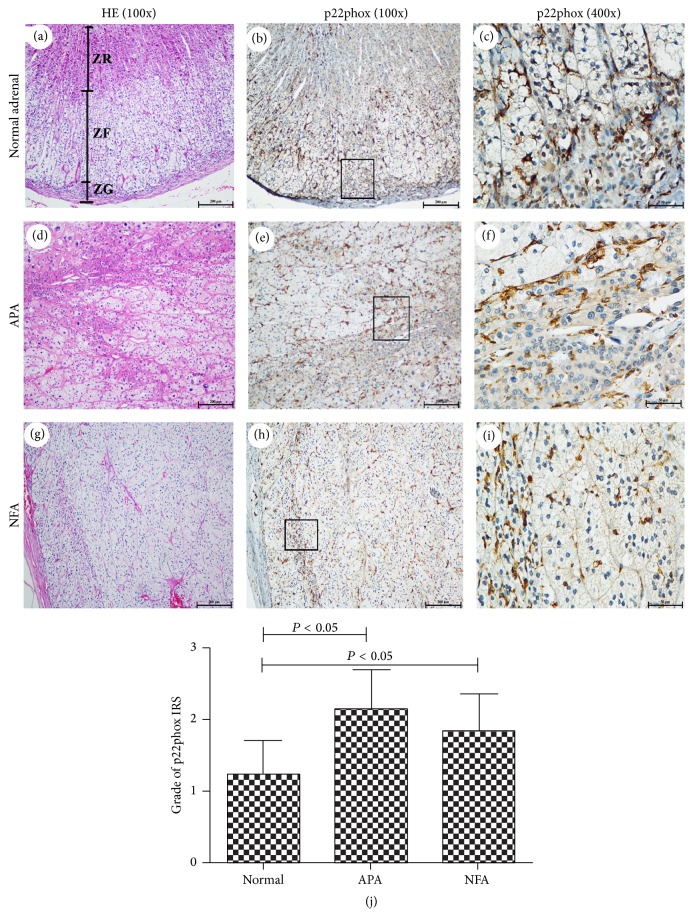
Pathological and immunohistochemical analyses of p22phox in normal adrenocortical tissue, APA, and NFA. (a–i) p22phox presents a gradient of expression in the entire adrenal cortex (100x), and its localization was restricted to the cell membrane (400x). (j) Graphical illustration of the statistical distributions of p22phox in normal adrenocortical tissue, APA, and NFA. The p22phox IRS was higher in APA compared to that in normal adrenal tissue.

To characterize more precisely the cellular composition of areas expressing Nox2 and p22phox, we investigated 11 normal adrenal tissues, 29 APA tissues, and 14 NFA tissues. The adrenal cortex is composed of three distinct morphological and functionally distinct zones: ZG, middle zona fasciculata (ZF), and inner zona reticularis (ZR). ZG is located underneath the capsule and produces mineralocorticoids. ZF, the thickest zone of the cortex, produces glucocorticoid hormones, whereas biosynthesis of sexual steroids takes place in the ZR. In this study, we did not use CYP11B2 as a specific ZG marker due in part to the 95% homology between CY11B2 and CYP11B1, which is the key rate-limiting enzyme for production of glucocorticoid hormones. Immunohistochemistry results demonstrated strong staining for Nox2 in the ZG cells (Figure 2(b)), where disabled-2 (Dab2) was found to be expressed (Figure 2(c)). Dab2 is a specific marker of the ZG of the adrenal cortex [17, 18]. Moreover, signals for Nox2 were also present in ZR cells in this study (Figure 2(b)). Consistent with the observed isoform specificity, Nox2 and Dab2 immunoreactivities were mapped in a subpopulation of steroidogenic cells representing ZG-like cells, especially under the capsule or around the vasculature in APA (Figures 2(e), 2(f), 2(h), and 2(i)). Nox2 expression was detected in the cytoplasm of ZG, ZR, ZG-like, and ZR-like cells. By contrast, p22phox was expressed in all the layers of the adrenal cortex (Figures 3(a) and 3(b)). Staining of p22phox was relatively restricted to the cell membrane of ZG, ZF, ZR, ZG-like, and ZF-like cells (Figures 3(b), 3(c), 3(e), 3(f), 3(h), and 3(i)). Although Nox2 and p22phox expression levels were weak in the normal adrenal cortex and NFA, immunostaining scores (IRS) of the two subunits revealed that they were obviously expressed in APA, especially Nox2 (Figures 2(p) and 3(j)). Additionally, in the semiquantitative analysis of Nox2 and p22phox by immunostaining, a statistically significant correlation was noted between the Nox2 IRS adjusted for tumor volume and baseline PAC (*R* = 0.503, *P* < 0.05), urinary aldosterone (*R* = 0.446, *P* < 0.05), and serum K^+^ (*R* = −0.550, *P* < 0.05). However, the IRS values for Nox2, p22phox, and p22phox adjusted for tumor volume were not significantly correlated with baseline PAC, urinary aldosterone, or serum K^+^ in APA (biological data of Nox2 and p22phox in APA was shown in Supplemental Table  2). The high IRS value for Nox2 in APA suggests that its expression may affect aldosterone biosynthesis.

### 3.3. Nox2 in Peritumoral Adjacent Tissue of APA

**Figure 4:**
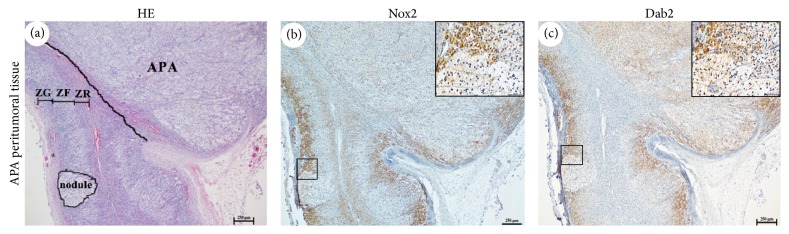
Morphological features and Nox2 expression in APA adjacent tissue. Adrenal hyperplasia is defined as a continuous distribution of adrenal ZG or extended focal thickness greater than 200 *μ*m. (a) In the APA adjacent tissue, ZG hyperplasia was demonstrated by HES. Adrenocortical nodule was surrounded by hyperplastic ZG cells and composed of ZF-like cells in APA. (b) Nox2 was expressed in the entire hyperplasic ZG and ZR cells, but not adrenocortical nodule. (c) Dab2 was expressed in the entire hyperplasic ZG cells. Bar, 250 *μ*m on low magnification (40x) and 50 *μ*m on high magnification (400x).

In normal adrenal tissues, HES staining is used to distinguish the different cortical cell layers. Interestingly, the present analysis of the morphology by HES staining revealed that the peritumoral tissues were not atrophic, showing a major increase of tissue remodeling and exhibiting hyperplastic changes compared with normal adrenal tissues, especially in the ZG layer (Figure 4(a)) which appeared continuous or presented extended focal thickening (>200 *μ*m) associated with Nox2 and Dab2 expression (Figures 4(b) and 4(c)). However, nodules without Nox2 expression were found in the hyperplasia. In addition, p22phox was detected in the nodular and all layers of the hyperplastic adrenal cortex in APA peritumoral tissues (data not shown).

Finally, pathological findings were revealed in 29 APA patients. APA with ZG hyperplasia in peritumoral tissue was present in 21 cases, while APA without ZG hyperplasia was found in 8 cases. We analyzed the clinical differences in APA with and without ZG hyperplasia, as shown in Table 2. Patients with ZG hyperplasia had statistically significantly higher preoperative baseline PAC compared with the patients without ZG hyperplasia. However, postoperative baseline PAC was not normal at one week after surgery in APA patients with ZG hyperplasia. In addition, the decline of blood pressure was greater at one week after partial adrenalectomy for systolic and diastolic blood pressure values in APA patients without ZG hyperplasia compared with ZG hyperplasia. Moreover, resolution of hypokalemia was achieved in all patients after resection. However, preoperative baseline PAC, tumor diameter, and postoperative serum K^+^ were not different between patients with different types of APA.

## 4. Discussion

In this study, we demonstrated that the Nox activity was higher in APA. The results suggest that Nox may represent a link between oxidative stress and aldosterone biosynthesis. Seven isoforms of Nox exist in mammals: Nox1, Nox2, Nox3, Nox4, Nox5, Duox1, and Duox2. Nox2 and p22phox, the catalytic core of the Nox, comprise an integral membrane heterodimer. Under resting conditions, Nox2 forms an inactive transmembrane complex with a smaller p22phox subunit that contributes to its maturation and stabilization. Furthermore, p22phox is thought to interact with other homologues of Nox2, including Nox1, Nox3, and Nox4. Therefore, RT-PCR was utilized to screen expression of these different isoforms in APA and normal adrenocortical tissues. The current study showed that* Nox1*,* Nox2*,* Nox4*,* Duox1*, and* p22phox* were expressed in normal adrenocortical tissues and APA. Furthermore, analysis by Q-PCR identified that* Nox2* and* p22phox* were especially abundant in APA compared with NFA and normal adrenocortical tissues. These results pointed towards a possible relationship between Nox2, p22phox, and APA. A significant correlation between* Nox2* mRNA and* CYP11B2* mRNA was detectable in APA, indicating that Nox2 was associated with aldosterone synthesis. However, no correlation was detected between* p22phox* mRNA and* CYP11B2* mRNA. We can speculate that p22phox can interact not only with Nox2 for aldosterone synthesis, but also with Nox2 homologues, Nox1 and Nox4, which were also expressed in the normal adrenal cortex and APA for other pathophysiological processes of adrenal diseases.

Western blot analysis revealed higher protein expression levels of Nox2 and p22phox in APA when compared with the control tissues. These results are in agreement with recent studies showing higher mRNA and protein levels of p22phox in APA compared with those in normal adrenal tissue [19]. Another study showed that increased oxidative stress in PA, characterized by increased serum levels of a Nox2-derived peptide, was reduced after adrenalectomy in APA [20]. Results of that study are also in line with our findings of high levels of Nox2-derived Nox in APA tissues.

Conversely, aldosterone can induce oxidative stress through genetic and nongenetic ways. Many studies have shown that aldosterone can increase Nox activity, resulting in ROS production and leading to myocyte apoptosis and glomerular diseases [9, 21]. The current study and previous studies showed that the interaction between Nox2 and aldosterone in the adrenal gland may be one reason for pathological autonomous aldosterone hypersecretion. Here, the relationship between clinical features and the different distribution patterns of Nox2 and p22phox in APA and the peritumoral adrenal cortex were also investigated.

However, the distribution and clinical relevance of Nox2 in APA differed from those of p22phox. Much of the work that has been reported in this field indicated that APA mainly consists of ZF-like, ZG-like, and hybrid cells, but very few ZR-like cells. Our combined results confirmed that Nox2 was found primarily in ZG cells with modest expression in ZR cells in normal adrenocortical tissues. Based on HES and Dab2 staining, Nox2 was mainly expressed in ZG-like cells of APA. Moreover, immunohistochemical analysis revealed that Nox2 immunoreactivity in APA was positively correlated with baseline PAC and negatively correlated with serum K^+^. However, the specific distribution of p22phox was not observed in the adrenal cortex and APA. Additionally, no correlation between p22phox immunoreactivity and baseline PAC was found. Immunohistochemical analysis confirmed again that Nox2-derived oxidase stress may be involved in regulating CYP11B2 expression and pathological aldosterone biosynthesis. The present data were consistent with a prior report showing that Nox2-derived oxidative stress enhanced aldosterone production in an adrenocortical tumor cell line [12].

Finally, the current study found that 72% of APA tissues presented adjacent hyperplasia and/or nodules. To gain insight into whether the hyperplasia or nodules are functional, we analyzed Nox2 localization by HES staining and Dab2 and compared the clinical outcomes at one week after partial adrenalectomy in APA patients with or without ZG hyperplasia. The results provided immunohistochemical evidence that, for some cases, aldosterone production was seen in the cortex adjacent to the APA. Often, the entire hyperplasic ZG strongly expressed Nox2, which was in line with the localizations of Dab2 and CYP11B2 reported by a recent study [22]. These findings suggest that APA and the adjacent ZG had acquired some ability for aldosterone synthesis, and therefore Nox2-derived oxidative stress may take part in the pathophysiology of autonomous aldosterone secretion after surgery. Interestingly, microscopic analysis of the cortex adjacent to the APA revealed that it contained small, multiple well-encapsulated nodules, which are often composed of admixtures of ZF-like cells and only pathologically identified, but Nox2 expression was not found in the nodules. Based on these findings, we propose that no aldosterone production may occur in the nodules, although one recent study suggested that the nodule may be the origin of new APA [23].

Furthermore, we compared the results with those of the adrenal cortex adhering to 29 APA tissues, and 21 of them presented ZG hyperplasia. Importantly, postoperative baseline PAC and blood pressure fell but did not reach normal levels in APA patients with ZG hyperplasia at one week after surgery. Therefore, we postulate that autonomous aldosterone production from the residual hyperplasia adjacent to the APA may lead to persistent postoperative hypertension. As noted earlier, a growing body of evidence suggests that adrenalectomy abolishes aldosterone hypersecretion and corrects serum K^+^ in most patients with APA at six months after surgery, but not hypertension, probably due to autonomous aldosterone production from the ZG hyperplasia of the residual adjacent tissue in APA and contralateral adrenal gland [24, 25]. Based on the current results and previous studies, we conclude that aldosterone production induced by Nox2-derived oxidative stress is related to persistent postoperative hypertension. Thus, one week after surgery may be the effective time to assess persistent postoperative hypertension according to the Nox2-derived oxidative stress. When hypertension persists after unilateral adrenalectomy for APA, histological examination for Nox2 and assessment of residual autonomous secretion of aldosterone may provide clinicians with objective information about treatment for persistent postoperative hypertension. In addition, our results emphasize the fact that APA patients after adrenalectomy will require short-term and long-term (even lifelong) follow-up, as well as regular control of blood pressure.

Taken together, these data suggest now that Nox2-derived oxidative stress may also play an important role in the pathology of adrenal steroid excess resulting in persistent postoperative hypertension. Prevention of Nox2-derived oxidative stress by targeting the enzymes responsible for their generation may be a more effective strategy for ameliorating oxidative stress than scavenging these highly reactive molecules once they are formed. Collectively, these current findings in conjunction with previous studies imply that Nox2, a frequent occurrence in aldosterone secretion, is a potential pharmacological target for persistent postoperative hypertension, thereby opening the way for therapeutic strategies.

## Supplementary Material

Supplemental Table 1. Primers and annealing temperature.
Supplemental Table 2. Correlation analysis of biological data with Nox2 IRS, p22phox IRS, Nox2 IRS×V and p22phox IRS×V in APA.

## Figures and Tables

**Table 1:** Clinical and endocrinologic features of patients with APA and NFA in this study.

Variable	APA (*n* = 29)	NFA (*n* = 14)	*P* value
Age (y)	44 ± 10	53 ± 14	<0.05
Sex (male/female)	9/20	6/8	NS
BMI (kg/m^2^)	22.0 ± 2.5	23.6 ± 3.6	NS
SBP (mmHg)	165 ± 14.1	136 ± 15.2	<0.01
DBP (mmHg)	100 ± 8.4	82 ± 9.7	<0.01
Duration of hypertension (y)	4.0 (0.5–7.0)	1.3 (0–10.0)	NS
Antihypertensive medication (grade)	2.0 (1.0–3.0)	1.0 (0–1.0)	<0.05
Diameter (cm)	1.6 (1.2–2.35)	2.9 (1.8–3)	<0.05
Volume (cm^3^)	2.6 (1.5–5.3)	8.1 (4.1–13.2)	<0.05
Baseline PRA (ng/mL·h^−1^)	0.1 (0.1–0.2)	2.3 (1.3–2.5)	<0.01
Baseline PAC (pg/mL)	609 (410–993)	113 (92.1–141.3)	<0.01
Baseline ARR (pg/mL per ng/mL·h^−1^)	6130 (2275–11600)	52 (40.6–81.2)	<0.01
Supine PRA (ng/mL·h^−1^)	0.11 (0.1–0.2)	0.1 (0.03–0.41)	NS
Supine PAC (pg/mL)	537 (387–739)	1185.5 (721–1436)	<0.05
Supine ARR (pg/mL per ng/mL·h^−1^)	488.2 (194.4–785.3)	768.6 (235–5452)	NS
Orthostatic PRA (ng/mL·h^−1^)	0.25 (0.065–0.37)	0.27 (0.14–0.41)	NS
Orthostatic PAC (pg/mL)	442 (327–655.2)	851.5 (549.8–1243.8)	<0.05
Orthostatic ARR (pg/mL per ng/mL·h^−1^)	295 (116.5–982.7)	260 (179.4–456.1)	NS
Serum K^+^ (mmol/L)	3.0 (2.8–3.4)	3.9 (3.6–4.2)	<0.01
Serum Na^+^ (mmol/L)	142.4 (140.5–144.8)	141.6 (140.5–143.6)	NS
Urinary K^+^ (mmol/24 h)	71.4 (55.4–97.2)	33.9 (25.4–41.1)	<0.01
Urinary Na^+^ (mmol/24 h)	151.7 (115.8–181)	92.0 (59.5–167.5)	<0.05
Urinary aldosterone (ug/24 h)	28.6 (20.4–51.2)	6.0 (4.8–8.8)	<0.01

BMI, body mass index; SBP, systolic blood pressure; DBP, diastolic blood pressure; PAC, plasma aldosterone concentration; PRA, plasma renin activity; ARR, aldosterone-to-renin ratio. Data are given as mean ± SD or median (interquartile range) as appropriate.

**Table 2:** Clinical and biological data of patients with or without ZG hyperplasia.

Variable	Patients with ZG hyperplasia (*n* = 21)	Patients without ZG hyperplasia (*n* = 8)	*P* value
Preoperative baseline, PAC, pg/mL	658 (421–1236)^#^	537 (387–879)^#^	NS
Postoperative baseline, PAC, pg/mL	329 (153–567)	27 (12–57)	<0.01
Preoperative baseline, PRA, ng/mL·h^−1^	0.1 (0.1–0.2)^#^	0.1 (0.0–0.4)^#^	NS
Postoperative baseline, PRA, ng/mL·h^−1^	1.2 (1.0–2.4)	2.7 (0.7–5.2)	<0.01
Preoperative baseline, ARR, pg/mL per ng/mL·h^−1^	488.2 (194.4–785.3)^#^	768.6 (235.1–5452)^#^	NS
Postoperative baseline, RR, pg/mL per ng/mL·h^−1^	126.5 (78.5–359.7)	4.5 (1.2–21.2)	<0.01
Preoperative-urinary, aldosterone, *µ*g/24 h	24.0 (19.3–40.2)	40.6 (26.9–54.4)^#^	NS
Postoperative-urinary, aldosterone, *µ*g/24 h	16.8 (8.5–28.1)	6.38 (1.0–14.6)	<0.05
Diameter, cm	1.6 (1.2–2.4)	1.8 (1.3–2.4)	NS
Preoperative SBP, mmHg	167.6 ± 13.7^#^	167.9 ± 21.2^*∗*^	NS
Postoperative SBP, mmHg	145.2 ± 7.5	123.6 ± 13.1	<0.01
Preoperative DBP, mmHg	101.6 ± 8.6^#^	95.9 ± 7.7^*∗*^	NS
Postoperative DBP, mmHg	94.7 ± 7.1	82.9 ± 5.5	<0.01
Preoperative serum K^+^, mmol/L	3.0 ± 0.5^#^	2.8 ± 0.3^*∗*^	<0.05
Postoperative serum K^+^, mmol/L	3.8 ± 0.3	3.87 ± 0.24	NS
SBP decline, mmHg	22.4 ± 13.1	44.25 ± 16.4	<0.01
DBP decline, mmHg	7.0 ± 11.8	13.0 ± 8.8	NS
Serum K^+^ rise, mmol/L	0.7 ± 0.5	1.1 ± 0.3	<0.05

PAC, plasma aldosterone concentration; PRA, plasma renin activity; ARR, aldosterone-to-renin ratio; SBP, systolic blood pressure; DBP, diastolic blood pressure. Data are given as mean ± SD or median (interquartile range), as appropriate. ^#^ *P* < 0.05 preoperatively versus postoperatively in patients with ZG hyperplasia; ^*∗*^ *P* < 0.05 preoperatively versus postoperatively in patients without ZG hyperplasia.
