# Supplementary material for: Role of Nox2 and p22phox in Persistent Postoperative Hypertension in Aldosterone-Producing Adenoma Patients after Adrenalectomy
Source: Int J Endocrinol. 2016 Feb 16;2016:2395634. doi: 10.1155/2016/2395634 (PMC4771902; doi:10.1155/2016/2395634)
Supplement: Supplementary file 1 — Supplemental Table 1. Primers and annealing temperature. Supplemental Table 2. Correlation analysis of biological data with Nox2 IRS, p22phox IRS, Nox2 IRS×V and p22phox IRS×V in APA. [file 2395634.f1.pdf]

Supplemental Table 1. Primers and annealing temperature

Gene	Sequence ID	Primer sequence (5'-3')	Annealing Temperature (℃)	Size (bp)
Nox1	NM_013955	F: CACTGACATCGTGACAGGTCTGAA	56	171
		R: CTGGAATATCGGTGACAGCATTTG		
Nox2	NM_000397	F: GCAATAACGCCACCAATCTGAAG	58	175
		R: GACTCATCCCAGCCAGTGAGGTA		
Nox3	NM_015718	F: GGTTCTGGCGATTTCAACAA	60	91
		R: AAGCCACGCTTTTTCATGTG		
Nox4	NM_016931	F: GTATGGAAGAGCCCAGATTCC	60	122
		R: GGTGACTGGCTTATTGCTCC		
Nox5	NM_024505	F: ACAGGAGAAGATGAACACATCTGGA	59	84
		R: GCATCCTCCTCGGCACTCAT		
Doux1	NM_017434	F: CTGGTCTTCAAGTCATCCGTCA	60	100
		R: TGTCAGCCAGCCACTCAAAC		
Doux2	NM_014080	F: GAGTTGGGTGACCAGCTGTCCTA	62	135
		R: TTCAGCCTGGCTCTTGCGTA		
P22phox	NM_000101	F: CAGTGGTACTTTGGTGCCTACTCC	60	90
		R: GGTGGAGCCCTTCTTCCTCT		
CYP11B2	NM_000498	F: GGAGCAGGGTTATGAGCAC	-	89
		R: GTGGTCCTCCCAAGTTGTAC		
GAPDH	NM_002046	F: GCACCGTCAAGGCTGAGAAC	55	138
		R: TGGTGAAGACGCCAGTGGA		

Table 2. Correlation analysis of biological data with Nox2 IRS, p22phox IRS, Nox2 IRS×V and p22phox IRS×V in APA

Variable	R（Nox2 IRS）	*P* value	R（Nox2 IRS×V）	*P* value	R（p22phox IRS）	*P* value	R（p22phox IRS×V）	*P* value
PAC	0.057	0.770	0.503	0.005	0.036	0.853	0.289	0.128
PRA	0.30	0.878	0.104	0.590	-0.107	0.582	-0.125	0.517
ARR	0.20	0.919	0.168	0.383	0.294	0.121	0.239	0.211
Uri-aldo	-0.042	0.830	0.466	0.011	-0.023	0.950	0.230	0.231
Serum K^+	0.019	0.923	-0.550	0.002	0.109	0.575	-0.015	0.939
Diameter	-0.026	0.285	0.775	0.001	-0.141	0.465	0.42	0.012

Abbreviations: PAC, plasma aldosterone concentration; PRA, plasma renin activity; ARR, aldosterone-to-renin ratio; Uri-aldo, 24h urinary aldosterone; IRS, immunoreactivescore; V, volume.
